# Simultaneous decompression of the orbital lateral wall and optic canal for fibrous dysplasia in early adolescence

**DOI:** 10.1186/s40064-016-2428-6

**Published:** 2016-06-14

**Authors:** Ryota Tamura, Tomoru Miwa, Yoshiaki Sakamoto, Maya Kohno, Kazuo Kishi, Kazunari Yoshida

**Affiliations:** Department of Neurosurgery, Keio University Hospital, 35 Shinanomachi, Shinjuku-ku, Tokyo, 160-8582 Japan; Department of Plastic and Reconstructive Surgery, Keio University Hospital, 35 Shinanomachi, Shinjuku-ku, Tokyo, 160-8582 Japan

**Keywords:** Early adolescence, Fibrous dysplasia, Optic canal, Orbital lateral wall

## Abstract

**Introduction:**

Patients with fronto-orbital fibrous dysplasia (FD) occasionally present fronto-orbital protrusion, exophthalmos, and visual acuity disturbance. Simultaneous management of these conditions has not been previously described.

**Case description:**

A-10-year-old female with fronto-orbital FD complained of left visual acuity disturbance. Head computed tomography showed compressed optic canal secondary to thickened bone. Decompression of the optic canal via the left frontotemporal extradural approach, opening of the lateral orbital wall, and dissection of the prominent zygoma were done simultaneously. The patient’s visual acuity disturbance and exophthalmos subsequently improved postoperatively.

**Discussion and evaluation:**

When optic canal decompression is performed by the fronto-temporal approach, opening of the lateral orbital wall can be done simultaneously to decrease the intraorbital pressure and to prevent exophthalmos. In addition, although aesthetic plastic surgery is not generally recommended during the growing phase (due to the possibility of recurrence), this approach can prevent skin loosening and adverse cosmetic outcomes.

**Conclusions:**

Aesthetic plastic surgery for fronto-orbital FD is recommended to prevent skin loosening. Opening of the lateral orbital wall should be performed when optic canal decompression is planned.

## Background

Fronto-orbital fibrous dysplasia (FD) is a relatively rare disease characterized by fronto-orbital protrusion, exophthalmos, and downward mobilization of the eyeball that leads to orbital dystopia. Patients with this condition may experience loss of visual acuity (Papay et al. 1995; Kaneshige et al. 2014; Abe et al. 2002, 2006; Ying et al. 2007; Yu et al. 1997). Treatment for progressive visual acuity disturbance associated with FD and aesthetic facial plastic surgery for FD during the growing phase are controversial approaches (Ying et al. 2007; Yu et al. 1997). We performed optic canal decompression and combined decompression of the orbital lateral wall to decrease the intraorbital pressure in a patient in early adolescence. This combined technique has not been previously reported. Visual acuity disturbance and exophthalmos improved in response to this combined technique. In addition, we discuss the necessity for aesthetic plastic surgery during the growing phase.

## Case description

### Onset and course

**Fig. 1:**
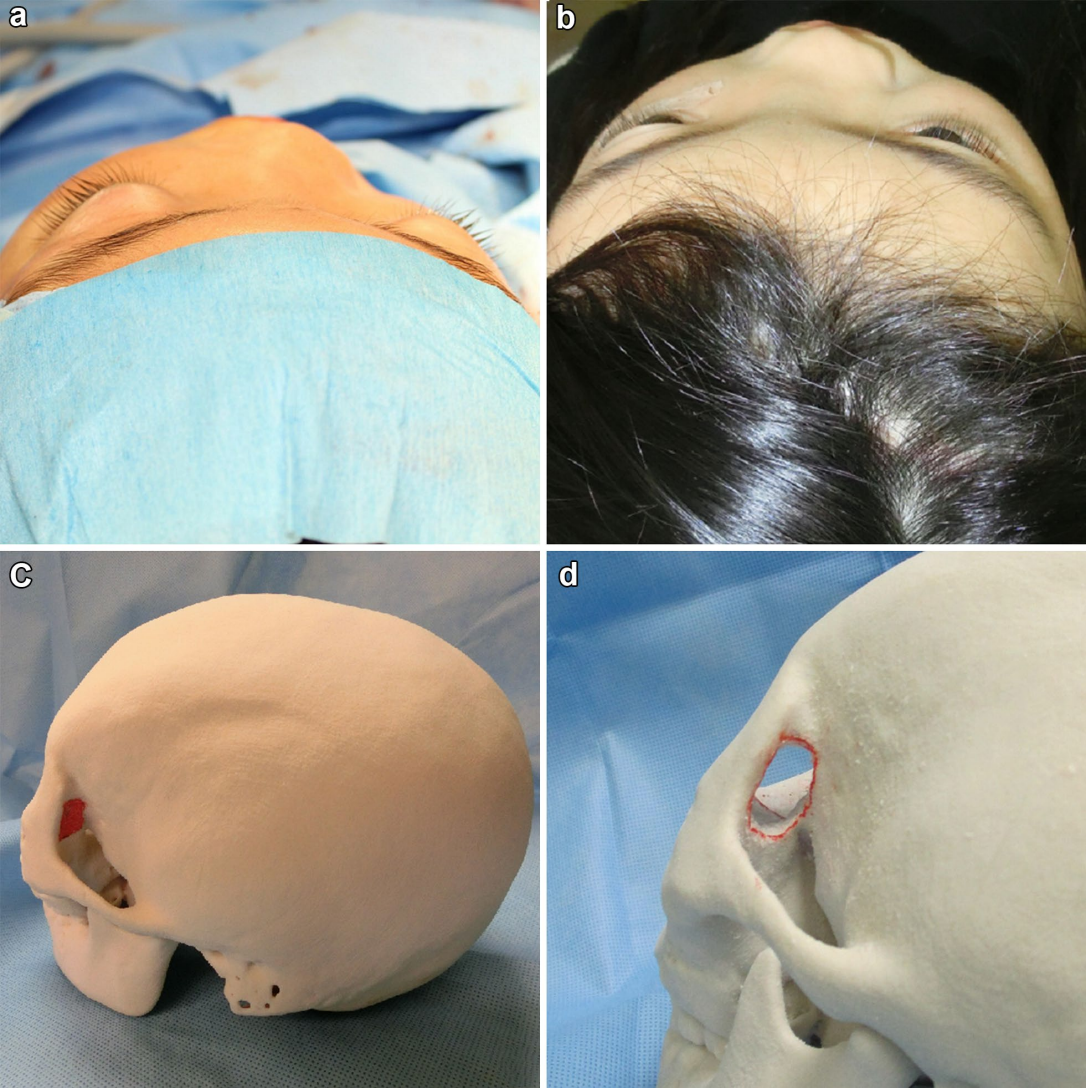
**a** This patient has a prominent *left* cheek and exophthalmos preoperatively. **b** *Left* exophthalmos is improved after the decompression of the lateral orbital wall. **c** Full size model. The area surrounded by the *red line* indicates the maximum size that can be filed off the lateral orbital wall. Decompression to this size is planned. **d** Model after decompression of the lateral orbital wall of maximum size. This is the same size as the actual operation

**Fig. 2:**
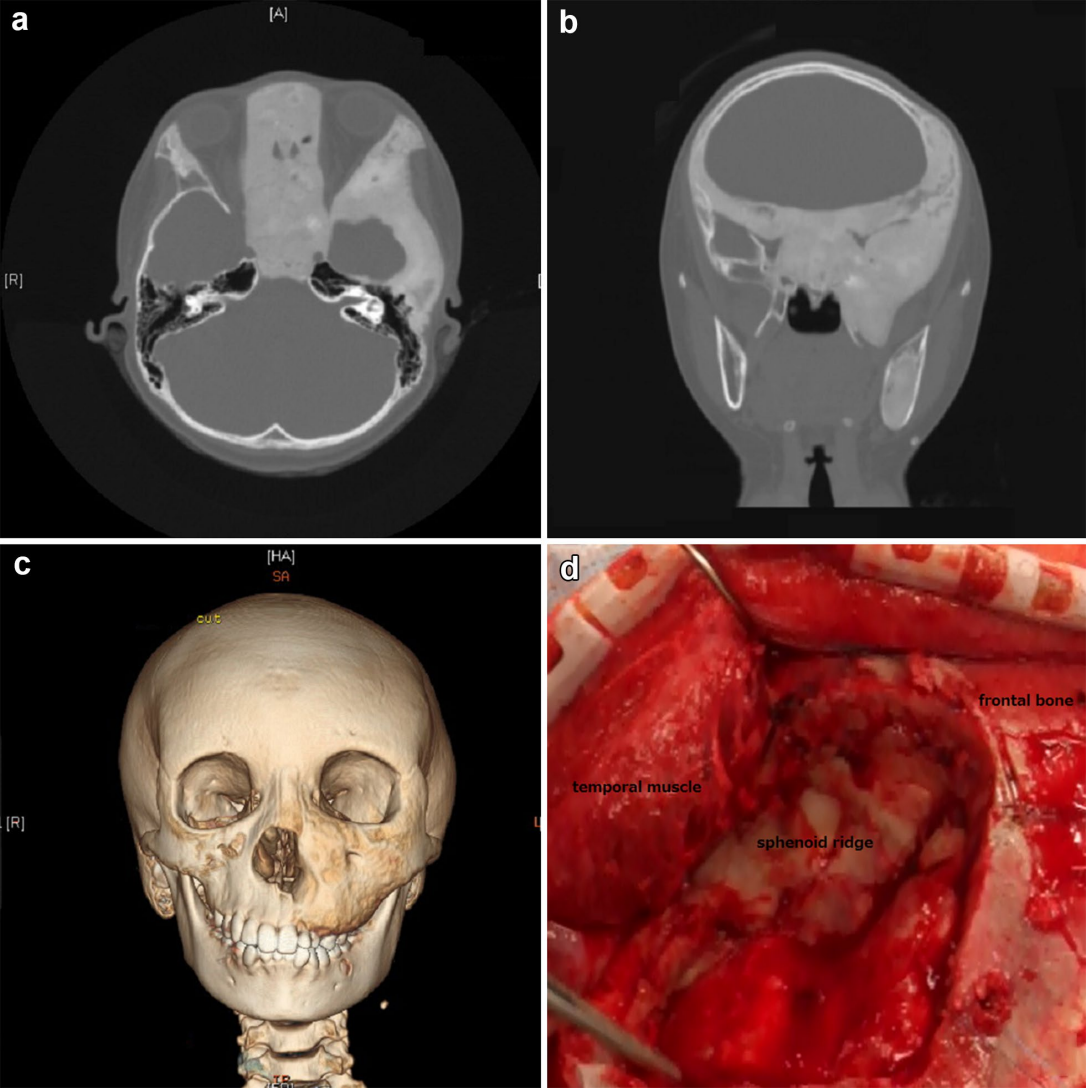
**a** High-resolution head axial CT shows extended bone hypertrophy of the *left* lesser wing of sphenoid bone and anterior clinoid process. **b** High-resolution head coronal CT shows narrowing of the *left* optic canal, which accounts for the visual acuity disturbance. **c** Bone 3 dimensional-CT shows prominence of the *left* facial bone and the marked difference relative to the *right* side. **d** The sphenoid bone is quite thick. It is difficult to identify the superior wall of the optic canal

A-10-year-old female was followed closely at another hospital for a 5-year history of FD of the skull base and nasal sinus bone caused by McCune–Albright syndrome. Fundus examination showed slight pale optic papilla (indicating chronic compressive neuropathy), but bilateral corrected visual acuity was normal at that time. She was noted to have enlargement of a Mariotte blind spot in the left visual field by Goldmann perimeter, which was first detected 3 years prior to presentation. The left visual field deficit worsened to hemianopia 2 years prior to presentation, and left visual acuity worsened to 0.1 [corrected visual acuity, 0.6 partial (p) × spherical (S) − 1.00 diopter (D)] 1 year prior to presentation. Left visual acuity rapidly worsened to 0.06 [0.3 × S − 1.00D: cylindrical (C) − 0.75D axis (Ax)145] at 6 months prior to presentation. Compression of the left optic canal was suspected. Left visual acuity was 0.05 (0.15 × S − 2.00D: C − 0.75D Ax 150) at first evaluation at our hospital. Right visual acuity was not disturbed. With regard to the visual field, left hemianopia was present (the same as 2 years prior to presentation). Pupil examination was normal with pupils equally responsive and reactive to light (PERRL) in each eye and with no relative afferent pupil defect (RAPD) Extraocular movements showed no abnormalities. Specialized eye examinations, including testing for color vision, showed no abnormalities. Physical examination revealed typical features of FD, such as prominent left cheek and exophthalmos (Fig. 1a) Distance from the lateral orbital rim to the corneal apex was measured using a Hertel exophthalmometer. The left side was 18 mm, and the right side was 14 mm the left side above average. Head computed tomography (CT) showed extended bone hypertrophy of the left facial bone and temporal bone (Fig. 2c). The lesser wing of sphenoid bone, anterior clinoid process, and nasal sinus were severely thickened (Fig. 2a, b). It had progressed over the last 4 years. The optic canal was compressed and was narrowed. Ophthalmic examination showed progressive worsening of visual acuity, slightly pale optic papilla and narrowed optic canal as shown by CT (indicating chronic compressive optic neuropathy).

### Operation

Plastic surgery was planned for left visual acuity disturbance, prominent cheek and exophthalmos. Therapeutic optic canal decompression was performed via a left frontotemporal extradural approach using a surgical navigation system (Medtronic, Tokyo, Japan). We drilled the superior wall of the superior orbital fissure and optic canal using a diamond bar under a microscope. This was performed with extra care as the orbital roof was affected and thickened by FD (Fig. 2d). In addition, dissection was continued toward the anterior clinoid process. Dissected bone was mixed in composition (soft and stiff). We identified the bulging appearance of the optic sheath, indicating that it was markedly compressed. There were no vascular complications during the operation.

**Fig. 3:**
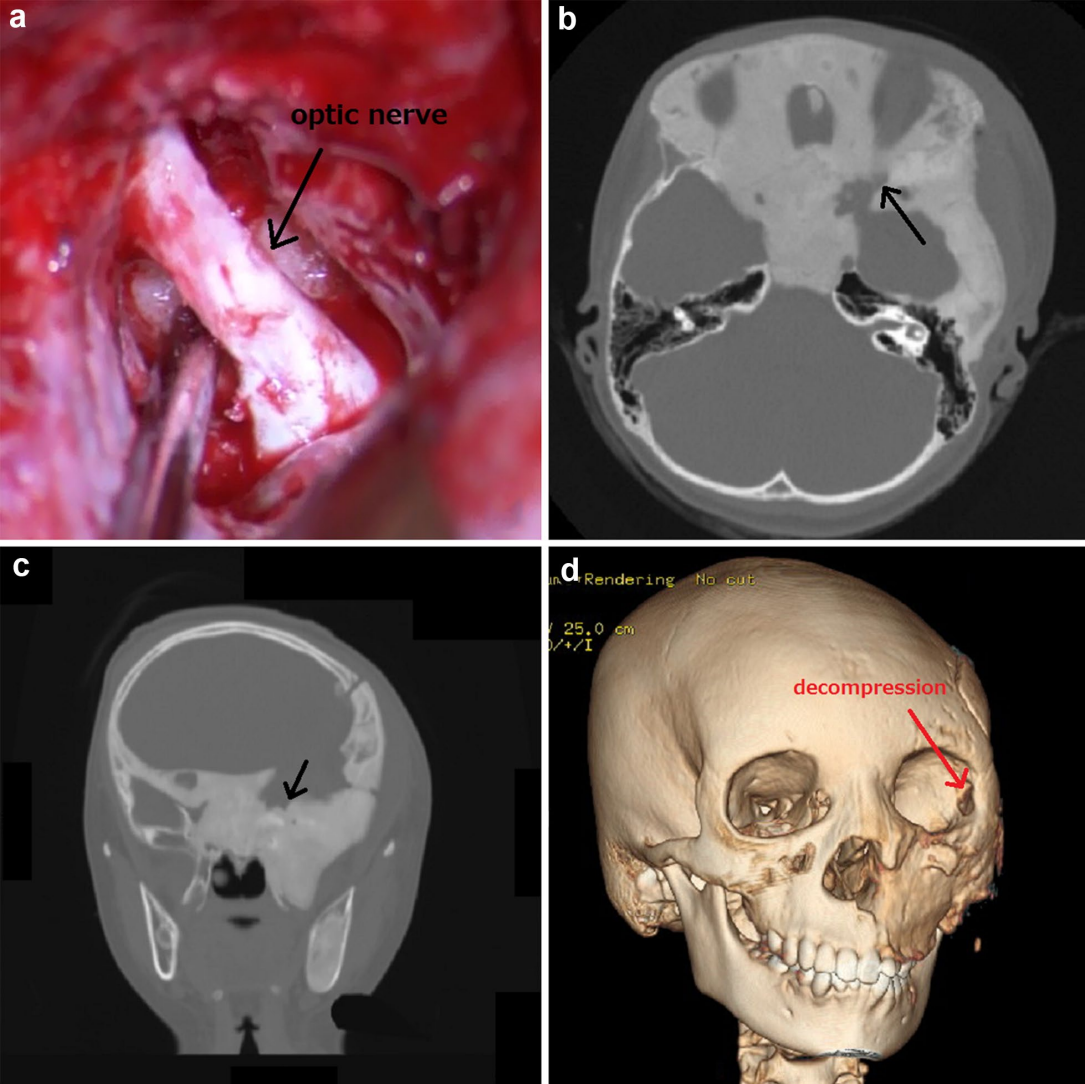
**a** The optic nerve is sufficiently decompressed via epidural decompression. **b**, **c** High-resolution head axial and coronal CT shows sufficient decompression of the optic canal (*arrows*, decompressed optic canal). **d** Bone 3 dimensional-CT shows improvement in the *left* facial bone. In addition, it shows decompression of the lateral orbital wall

We dissected the hypertrophic bone around the optic sheath as much as possible. The size of the decompressed canal was 20 mm longitudinally and 12 mm horizontally. Increased mobility of the optic sheath was identified (Fig. 3a). Next, a plastic surgeon made a blowout hole (2 × 2 cm) using a cutting bar on the lateral orbital wall to decrease the orbital tissue pressure (Figs. 1c, d, 3d). At that time, we needed to prevent tissue in the orbit. Through the tunica conjunctiva and oral vestibule, we dissected the prominent cheek using a rasp. We identified the infraorbital foramen and preserved the maxillary nerve. This resulted in the left cheek appearing equal in size to the right cheek.

### Postoperative course

Postoperative CT revealed satisfactory decompression of the left optic canal (Fig. 3b, c) Left visual acuity gradually improved (0.125p × S − 2.5D: C − 0.75D Ax 15) by 30 days after the operation. Her formerly prominent left cheek and exophthalmos became practically unnoticeable (Fig. 1b).

## Discussion and evaluation

Fronto-orbital FD sometimes causes disturbance of ocular movement as well as deterioration and disturbance of visual acuity (Papay et al. 1995; Kaneshige et al. 2014; Rahman et al. 2009). Therapeutic decompression is an accepted approach for this condition, especially for the cases with progressive gradual visual disturbance and within 1 week of a sudden visual loss secondary to FD (Ying et al. 2007; Yu et al. 1997).

However, the efficacy of prophylactic decompression is controversial. In fact, some studies suggested that even if narrowing of the optic canal is noticed, visual acuity is not necessarily affected. In addition, decompression of the optic canal, especially for FD, carries a risk of iatrogenic visual impairment, even when treated by experienced neurosurgeons, as reported by Kaneshige et al. (2014). The operative difficulty is increased by the thickness of bone with abnormal anatomy of the frontal skull base and the surrounding of the anterior clinoid process. It is also difficult to secure a clear working space because the diploe tends to bleed in cases of FD. Therefore, several authors have suggested working from the normal tissue towards the diseased tissue and avoiding drilling in the area of the optic canal (to avoid vascular incidents) (Janice et al. 2002; Ying et al. 2007; Yu et al. 1997). In addition, use of a navigation system can help prevent iatrogenic visual loss.

Recurrent FD after optic nerve decompression has not been widely reported (Rahman et al. 2009; Abe et al. 2002, 2006; Ying et al. 2007; Yu et al. 1997). However, Kusano et al. (2009) suggested that FD related to McCune–Albright syndrome tends to recur after decompression of the optic canal and is associated with poor outcomes. As a result, management of visual acuity disturbance caused by FD with involvement of the optic canal remains controversial. We believe that optic canal decompression for FD is beneficial because the visual acuity of several patients has improved after optic nerve decompression. In addition, opening of the lateral orbital wall can decrease the intraorbital pressure and prevent exophthalmos. When optic canal decompression is performed via the fronto-temporal approach, combined opening of the lateral orbital wall can be done simultaneously in the same operative field. A required step is separating the periorbita from the orbital lateral wall, and the lateral wall opened using a cutting bar to protect the periorbita. Generally, after complete osteotomy of the orbital lateral wall, the orbital edge should be fixed with a titanium plate. However, as artificial materials should not be used, in the present case we saved a 5-mm edge the orbital edge when the lateral wall of the orbita was opened because. The maximum size of decompression is up to the greater sphenoid wing. Orbital lateral wall decompression is said to be effective for ophthalmopathy. Decompression could improve the exophthalmos by 5 mm on average (Goldberg et al. 1998, 2000; Leone et al. 1989).

Visual acuity disturbance of this patient is probably caused by increased orbital pressure and optic canal stenosis. Therefore, opening of the lateral orbital wall via decompression and optic canal decompression should be performed at the same time. In addition, lateral orbital wall decompression has a beneficial cosmetic effect in patients with exophthalmos. Generally, aesthetic plastic surgery during the patient’s growing phase should not be done, because facial prominence has been thought to be generally progressive. This disease tends to resolve after puberty, so plastic surgery is recommended only in adults, because if symmetrical reconstruction of the prominent area was done, there is a high risk of recurrence in a patient in the growing phase. (Ying et al. 2007; Yu et al. 1997). However, if aesthetic plastic surgery is performed during adulthood, skin loosening is often identified. In addition, FD related to McCune–Albright syndrome often shows rapid progression and continues after early adolescence (Kusano et al. 2009), resulting in worsening skin loosening. This can cause significant adverse social effects for younger patients with FD (e.g., they can be targets for social bullying based on their appearance). Therefore, patients with McCune–Albright syndrome should undergo aesthetic plastic surgery, even during the growing phase. We recommend additional early stage reconstruction for such patients, especially if there is a plan to decompress the optic canal to address progressive gradual visual disturbance.

## Conclusions

If a patient has FD with exophthalmos and visual acuity disturbance caused by optic canal stenosis, simultaneous decompression of the orbital lateral wall and optic canal should be performed for the cause of that they are able to perform at same pterional skin incision. In this manner, exophthalmos and visual acuity disturbance can improve simultaneously. Aesthetic plastic surgery is recommended for orbito-frontal FD caused by McCune–Albright syndrome in the growing phase to prevent skin loosening.
